# Hypomorphic A20 expression confers susceptibility to psoriasis

**DOI:** 10.1371/journal.pone.0180481

**Published:** 2017-06-28

**Authors:** Anri Aki, Miyuki Nagasaki, Barbara Ann Malynn, Averil Ma, Takashi Kagari

**Affiliations:** 1 Biologics & Immuno-Oncology Laboratories, Oncology Function, R&D Division, Daiichi Sankyo Co., Ltd., Tokyo, Japan; 2 Department of Medicine, University of California San Francisco, San Francisco, CA, United States of America; Johns Hopkins School of Medicine, UNITED STATES

## Abstract

Psoriasis is a common inflammatory skin disease that affects approximately 1% of the population worldwide. Tumor necrosis factor-alpha-induced protein 3 (TNFAIP3) gene polymorphisms have been strongly associated with psoriasis susceptibility. In this study, we investigate how TNFAIP3, also known as A20, may regulate psoriasis susceptibility. We found that haplo-insufficient A20^+/-^ mice develop severe toll-like receptor (TLR)-induced skin inflammation compared to wild type mice owing to amplified production of interleukin (IL)-17 and IL-23. Examination of TNFAIP3 mRNA expression in skin biopsies from patients with psoriasis revealed reduced expression in both involved and uninvolved skin. Our results demonstrate the clinical importance of reduced dermal expression of A20 in psoriasis and suggest that A20 restriction of the IL-23/17 axis protects against psoriasis.

## Introduction

Psoriasis is a common immune-mediated skin disease that is caused by complex factors including the environment and genetic background. Recently, a genome wide association study of psoriasis cases revealed susceptibility loci with confirmed association to the disease, including human leukocyte antigen (HLA)-C, three genes involved in IL-23 signaling (IL23A, IL23R, IL12B), two genes acting downstream of tumor necrosis factor-alpha (TNFα) and regulating nuclear factor-kappa B (NF-κB) signaling (TNIP1, TNFAIP3), and two genes involved in the modulation of T helper 2 (Th2) immune responses (IL4, IL13) [1]. These results implicate these three pathways in psoriasis pathogenesis and suggest that genetic susceptibility could partially explain heterogeneity in treatment responses to TNFα blockade among patients with psoriasis. In this regard, a recent report showed that TNFAIP3 gene single nucleotide polymorphisms (SNPs) were associated with response to TNFα blockade in psoriasis [2].

The TNFAIP3 gene was originally identified as a TNF-inducible gene, which encodes the A20 protein, a negative feedback inhibitor of TNF-induced NF-κB signaling [3]. A20 is a potent anti-inflammatory protein, whose loss leads to spontaneous inflammation and perinatal lethality [4]. This lethality is due to A20 capacity to restrict MyD88-dependent TLR signals [5, 6]. A20 also inhibits nucleotide-binding oligomerization domain-containing protein 2 (NOD2), antigen receptor, and cluster of differentiation 40 (CD40)-induced signals [7–9]. A20 functions in a complex biochemical manner, exhibiting deubiquitinating capacity, ubiquitin binding, and E3 ligase activities that inhibit ubiquitin-dependent NF-κB signals [10–13]. A20 also inhibits inflammasome activity and cell death [14–16], providing an additional potential mechanism that prevents disease.

TNFAIP3 gene SNPs are strongly associated with psoriasis, and at least one of these SNPs leads to reduced A20 expression in cell lines [17, 18]. Epidermis-specific knockout of A20 in mice caused keratinocyte hyperproliferation without signs of spontaneous inflammation [19]. Hence, the mechanisms by which A20 regulates dermal immune homeostasis and psoriasis susceptibility remain unclear. The purpose of this study is to investigate how TNFAIP3 may regulate psoriasis susceptibility.

## Materials and methods

### Mice

C57BL/6J A20^+/-^ and A20^+/+^ littermate control mice were generated at the University of California, San Francisco, and bred in Charles River Laboratories Japan, Inc., Yokohama, Kanagawa, Japan, as previously described [4]. Upon arrival, mice were randomized into experimental groups. They were housed 4 to 5 per cage with free access to food and water under specific pathogen free conditions. Mice were maintained at constant temperature and humidity (21°C–25°C and 45–65%, respectively) under a 12 h light/dark cycle, and used at 10–17 week-old. This study was approved by the Institutional Animal Care and Use Committee of Daiichi Sankyo Co., Ltd. (Permit Numbers: A1200892, A1300186, A1402800, and A1403341). All surgeries were performed under isoflurane anesthesia, and all efforts were made to minimize suffering. All mice were sacrificed by aortic puncture and exsanguination under isoflurane anesthesia.

### Experimentally induced psoriasis-like dermatitis

Imiquimod (IMQ) treatments were performed as previously described [20]. Twenty milligrams Beselna cream (Mochida Pharmaceutical Co., Ltd., Tokyo, Japan) containing 5% IMQ or cream control (Vaseline, Kosakai Pharmaceutical Co., Ltd., Tokyo, Japan) was applied to both ears of the mice daily for 3 or 8 days. Ear thickness was measured every day with a dial thickness gauge, and mice were sacrificed at Day 3 or 8. Ear thickness was measured each day immediately before treatment, starting at Day 0.

### Intradermal IL-23 injections

Cutaneous inflammation was induced by injecting the ears of anesthetized mice every day with 20 μL phosphate-buffered saline (PBS) containing 1 μg bovine serum albumin (BSA) or 1 μg IL-23 (R&D Systems, MA, USA) using a 30-gauge needle attached to a Hamilton syringe every day for 4 days. Ear thickness was measured each day immediately before injection, starting at Day 0.

### Cell preparation from draining lymph nodes

Mice were sacrificed, and infra-auricular lymph nodes were isolated. Lymph nodes were embedded in ice-cold RPMI 1640 medium (Thermo Fisher Science K.K., Yokohama, Kanagawa, Japan) containing 10% fetal bovine serum (FBS) and 1% penicillin / streptomycin (P/S) (100 units/mL penicillin /streptomycin, Thermo Fisher Science K.K.), teased into a single cell suspension by pressing with the plunger of a 2.5-mL syringe, and filtered by a 70-μm cell strainer. Centrifugation was performed for 5 min at 870×*g*, and supernatants were discarded. Pellets were resuspended in RPMI 1640 medium containing 10% FBS and 1% P/S, and cell count and viability analysis were performed.

### Cell preparation from spleens

Mice were sacrificed and the spleens were dissociated. The spleens were embedded in a 6-cm Petri dish filled with 5 mL Hanks’s balanced salt solution (HBSS) Ca^2+^/ Mg^2+^ (Thermo Fisher Science K.K.) containing 2 mg/mL collagenase D (Roche Diagnostics K.K., Tokyo, Japan) and 50 μg/mL DNase I (Roche Diagnostics K.K.). Using a 1-mL syringe with 26G × ½^”^ needle, the digestion mix was injected in the spleen gently. After permeation of the digestion mix, spleens were minced into small pieces in approximately 1 × 1 mm blocks using a needle and incubated for 30 min at 37°C in a humidified incubator containing 5% CO_2_. After incubation, cells were filtered with a 100-μm cell strainer, and the Petri dish and cell strainer were washed with 5 mL HBSS Ca^2+^/Mg^2+^.

For lysis of red blood cells, cells were centrifuged for 3 min at 870 × *g*, and the supernatants were aspirated. 1.5 mL/spleen red blood cell lysing buffer (Sigma-Aldrich Japan Inc., Tokyo, Japan) were added to the pellets, incubated for 1 min at room temperature, and neutralized by 5 mL/spleen RPMI 1640 containing 10% FBS. To wash cells, the cells were centrifuged for 3 min at 870 × *g*, and supernatants were aspirated. Cells were resuspended in RPMI 1640 containing 10% FBS and counted using a cell counter (Vi-CELL™ XR, Beckman Coulter, Inc.,CA, USA).

### TLR7/8 stimulation *in vitro*

Splenocytes were stimulated by IMQ (1 μg/mL) in RPMI 1640 containing with 10% FBS and 1% P/S for 0, 4, 7, and 24 h, and the cell culture media were collected. The amount of IL-1α, IL-1β, IL-2, IL-3, IL-4, IL-5, IL-6, IL-9, IL-10, IL-12 (p40), IL-12(p70), IL-13, IL-17A, Eotaxin, granulocyte colony-stimulating factor (G-CSF), granulocyte-macrophage (GM)-CSF, interferon-γ (IFN-γ), keratinocyte chemoattractant (KC), macrophage chemoattractant protein-1 (MCP-1), macrophage inflammatory protein-1α (MIP-1α), MIP-1β, regulated upon activation, normal T cell expressed and secreted (RANTES), and TNFα were measured by a Bio-Plex Pro Mouse Cytokine 23-plex Assay (Bio-Rad Laboratories, Inc., CA, USA)

### Flow cytometry analysis

Cell surface-staining was performed on mice blood, spleens, isolated cells from ears, or draining lymph nodes. Isolated cells were resuspended in fluorescence-activated cell sorting (FACS) buffer (PBS containing 2% FBS and 0.1% NaN3) and Fc block (12.5 μg/mL; anti-mouse CD16/32; Nippon Becton Dickinson Company, Ltd., Tokyo, Japan) before incubation with fluorochrome-conjugated antibodies (3.5 μg/mL; CD4, CD3, CD19, CD11b, Gr-1, IL-17, T cell receptor γδ(TCRγδ) or TCRβ; BD and NK1.1; Thermo Fisher Scientific K.K.). For intracellular staining, cells were resuspended in RPMI 1640 containing 10% FBS and stimulated with phorbol 12-myristate 13-acetate (10 ng/mL; Sigma-Aldrich Japan, Inc.) and A23187 (100 ng/mL; Sigma-Aldrich Japan, Inc.). To obtain a better signal, Fixation and Permeabilization Solution Kit and BD GolgiPlug kit (Nippon Becton Dickinson Company, Ltd.) were used according to the manufacturer protocols. Flow cytometry was performed using FACSCalibur (Nippon Becton Dickinson Company, Ltd.). Data were further analyzed by FlowJo 7.6.5 software (Tree Star, Inc., OR, USA).

### Measurement of cytokine expression in ears

One ear of sacrificed mice was cut and frozen in liquid nitrogen. Ear homogenate samples were prepared with Bio-Plex Cell Lysis Kit (Bio-Rad Laboratories, Inc.). Briefly, the ears were rinsed with cell wash buffer provided as a part of the kit and minced in ice-cold lysing solution with 20 mM phenylmethylsulfonyl fluoride, and homogenized using a Physcotron (Microtec co., Ltd., Funabashi, Chiba, Japan), and homogenized in ice. The homogenates were centrifuged for 4 min at 4,500 × *g*, and the supernatants were centrifuged for 10 min at 5,000 × *g*. The supernatant protein concentrations were measured, adjusted to 0.5 mg/mL, and subjected to Bio-Plex Pro Mouse Cytokine 23-plex assay for murine IL-1α, IL-1β, IL-2, IL-3, IL-4, IL-5, IL-6, IL-9, IL-10, IL-12 (p40), IL-12(p70), IL-13, IL-17A, eotaxin, G-CSF, GM-CSF, IFN-γ, KC, MCP-1, MIP-1α, MIP-1β, RANTES, and TNFα according to the manufacturer protocol. The concentration of the total protein in the supernatants was measured by using a protein assay dye reagent (Bio-Rad Laboratories, Inc.) calibrated against the concentration of BSA. The concentration of cytokines and chemokines was expressed in picograms per milligram of protein.

### Patient data

A microarray dataset from patients with psoriasis or healthy controls was downloaded from GEO (accesion no. GSE14905 [21]). There was another dataset from patients with pasoriasis, but we did not use it to estimate the expression level of TNFAIP3 among patients or healthy controls because of its very low expression level of TNFAIP3 and IL-17A (GEO accession no. GSE13355 [1]).

### Statistical analysis

All data were expressed as means ± standard error of the mean (SEM). The statistical significance of differences between groups was tested by the Student’s *t*-test, and statistical significance of differences between more than two groups were tested by the Dunnett’s test or Steel’s test. P-values less than 0.05 were considered significant. The statistical significance of differences between the scores of two groups was tested by the Wilcoxon Rank-Sum test.

## Results and discussion

### Aggravated IMQ-induced ear inflammation in hypomorphic A20^+/-^ mice

**Fig 1:**
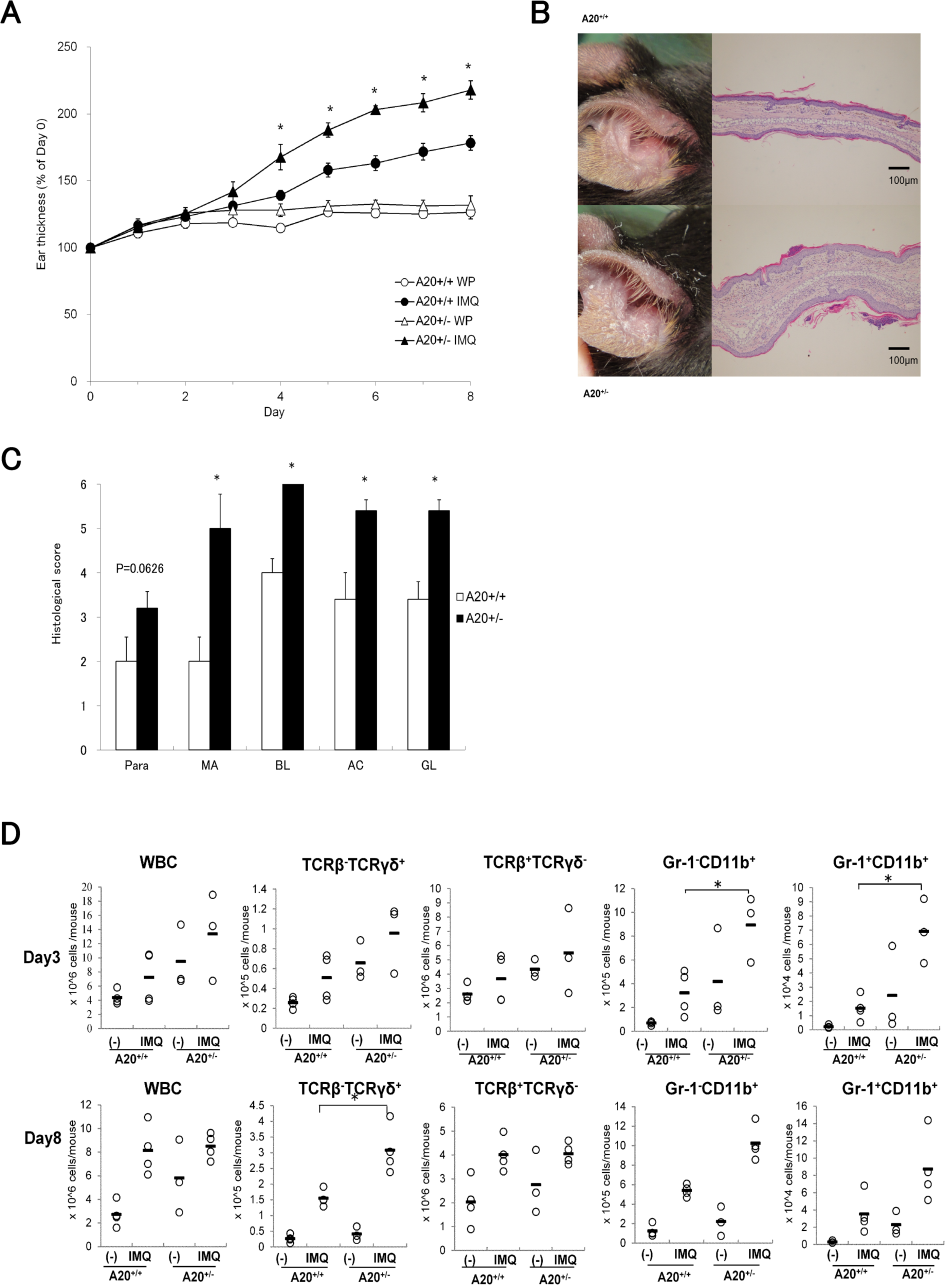
Deficiency of A20 aggravates ear inflammation in an IMQ-induced psoriasis model. (A) Thickness of IMQ or control cream white petrolatum (WP)-treated ears in male A20^+/-^ and A20^+/+^ littermate mice. Results are representative of three experiments. Error bars represent SEM; N = 3 (control) or 5 (IMQ-treated) for each group,*p < 0.05 compared to IMQ-treated A20^+/+^ mice by Student’s *t*-test. (B) Appearance of inflamed ears of male A20^+/-^ and A20^+/+^ littermate mice and hematoxylin and eosin (H&E)-stained sections of IMQ- or WP-treated ears in indicated genotypes at Day 8 (N = 5, 100 X magnification). (C) Histological scores of the sum of both ears on the basis of histologic findings; parakeratosis (Para), microabscess (MA), basal layer cell proliferation (BL), acanthosis (AC), and hypergranulosis (GL) are shown for male A20^+/−^ and A20^+/+^ littermate mice 8 days after IMQ treatment. Scoring was performed as follows: 1, mild; 2, moderate; 3, severe. Error bars represent SEM; N = 5 for each group. *p < 0.05 compared to IMQ-treated A20^+/+^ mice by Wilcoxon Rank-Sum test. (D) Numbers of white blood cells (WBCs), TCRβ^+^TCRγδ^-^ cells, TCRβ^-^TCRγδ^+^ cells, Gr-1^-^CD11b^+^ cells, and Gr-1^+^CD11b^+^ cells from ear-draining lymph nodes for indicated days in IMQ-treated or WP-treated male A20^+/-^ and A20^+/+^ littermate mice. Results are representative of two experiments. Circles represent individual data, and horizontal bars represent the mean of each group. N = 3 or 4 for each group, *p < 0.05 compared to IMQ-treated A20^+/+^ mice by Student’s *t*-test.

Reduced A20 protein expression and aberrant signaling responses have been observed in heterozygous A20FL/+ CD19-Cre mice and A20FL/+ CD11c-Cre mice [9, 22]. We examined whether reduced A20 expression in A20^+/-^ mice affected their susceptibility to topical treatment with IMQ. In our A20^+/-^ mice, reduced expression of TNFAIP3 compared to A20^+/+^ mice (S1 Fig) was confirmed. IMQ causes a psoriasis-like condition in humans and psoriasis-like lesions in mice [20]. Treatment of mice with IMQ caused increased ear swelling and edema in A20^+/-^ mice compared to that in A20^+/+^ mice (Fig 1A). Ears of IMQ-treated A20^+/-^ mice were covered with psoriasis-like scales after 8 days of IMQ treatment (Fig 1B). Histologic examination of skin sections revealed that A20^+/-^ mice increased typical histologic findings in human psoriasis such as parakeratosis, microabscess, basal layer cell proliferation, acanthosis, and hypergranulosis (Fig 1C).

To elucidate the inflammatory factors contributing to increased ear swelling in A20^+/-^ mice, we performed FACS analyses of the cells in draining lymph nodes. In lymph nodes, the numbers of pro-inflammatory TCRβ^-^TCRγδ^+^ γδT cells, Gr-1^-^CD11b^+^ macrophages, and Gr-1^+^CD11b^+^ neutrophils increased in IMQ-treated A20^+/-^ mice compared to that in A20^+/+^ mice (Fig 1D). Recently, γδT cells and neutrophils were reported to be a major source of IL-17 production in psoriatic lesions and to form a chronic inflammation loop in psoriatic skin [23, 24]. This result suggests that A20 controls direct and/ or indirect recruitment of γδT cells, macrophages, and neutrophils into draining lymph nodes through TLR7/8 signal and may restrict formation of the psoriatic environment.

**Fig 2:**
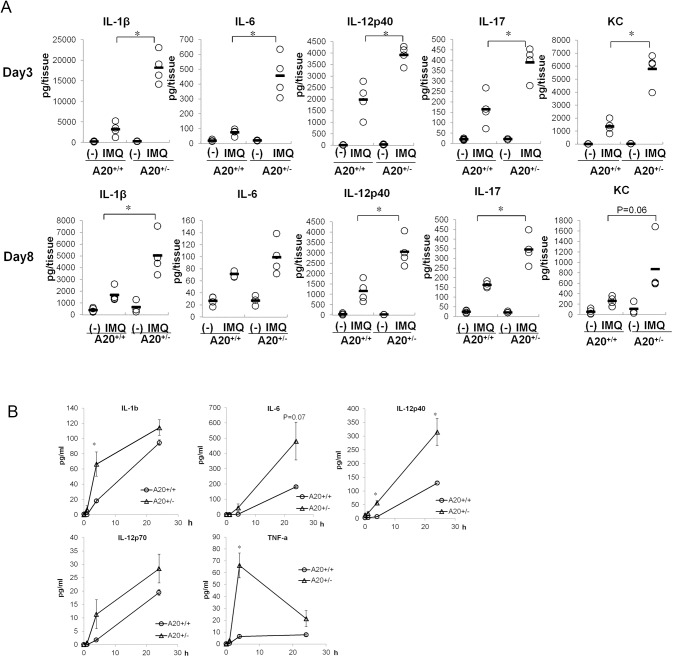
A20 restricts psoriasis-related inflammatory cells responses by TLR7/8 stimulation. (A) Cytokine concentration in ears of male A20^+/-^ and A20^+/+^ littermate mice treated with WP or IMQ for 3 or 8 consecutive days. Circles represent individual data, and horizontal bars represent the mean for each group. N = 3 or 4 for each group, *p < 0.05 compared to IMQ-treated A20^+/+^ mice by Student’s *t*-test (B) Time course of cytokine production in splenocytes stimulated with IMQ (1 μg/mL). Splenocytes were obtained from non-treated male A20^+/-^ and A20^+/+^ littermate mice, stimulated with IMQ for the indicated time, and the produced cytokines in the culture supernatant were mesured. Data are representative of two experiments. Error bars represent SEM; N = 3 for each group, *p < 0.05 compared to cytokine production in A20^+/+^ splenocytes by Student’s *t*-test.

To determine the effect of A20 on TLR7/8-induced cytokine production, we measured cytokine concentrations in ear extracts after repeated IMQ treatments. The psoriasis- related cytokines IL-1β, IL-6, IL-12p40, IL-17, and KC increased in ears of repeated IMQ-treated A20^+/-^ mice compared to that in control mice even at the early stage of induction (Fig 2A, S1 Table). To elucidate whether A20 restricted TLR7/8-mediated cytokine production, we tested TLR7/8 agonist on splenocytes obtained from A20^+/+^ and A20^+/-^ mice. Prior to this experiment, we confirmed the population of splenocytes in each genotype of mice. There were no notable differences in the proportions of CD3^+^, CD19^+^, Gr-1^+^CD11b^+^, and Gr-1^-^CD11b^+^cells between A20^+/+^ and A20^+/-^ mice (S2 Fig). As a result, reduction in A20 expression caused notably increased production of IL-1β, IL-6, TNFα, and IL-12p40 in splenocytes (Fig 2B, S2 Table). This phenomenon suggests the augmented sensitivity to TLR7/8 ligand by modulating TLR7/8 pathway molecules or increased numbers of TLR7/8 receptor-expressing cells or TLR7/8 receptor expression itself. We also generated A20^+/−^ bone marrow chimeras by reconstituting lethally irradiated A20^+/+^ recipient mice with A20^+/−^ bone marrow cells to elucidate whether immune cells were responsible for aggravated dermatitis by reduced expression of A20. We confirmed that IMQ caused increased ear swelling in A20^+/−^ bone marrow chimeras compared to that in A20^+/+^ bone marrow chimeras; however, the extent of the aggravation of ear swelling in A20^+/-^ bone marrow chimeras was not as severe as that observed with systemically reduced expression of A20 (Fig 1A, S3 Fig). This suggested that reduced expression of A20 aggravated skin lesion through not only immune cells but also non-immune cells such as keratinocytes. Previously, Lippens et al. reported that epidermis-specific A20-knockout mice developed keratinocyte hyperproliferation without skin inflammation [19]. Further studies using these keratinocyte-specific A20-knockout mice would be helpful for a better understanding of the cells involved in the dermatitis induced by A20-reduced expression.

### IL-12/23p40 and IL-17 are involved in psoriasis severity in A20^+/-^ mice

**Fig 3:**
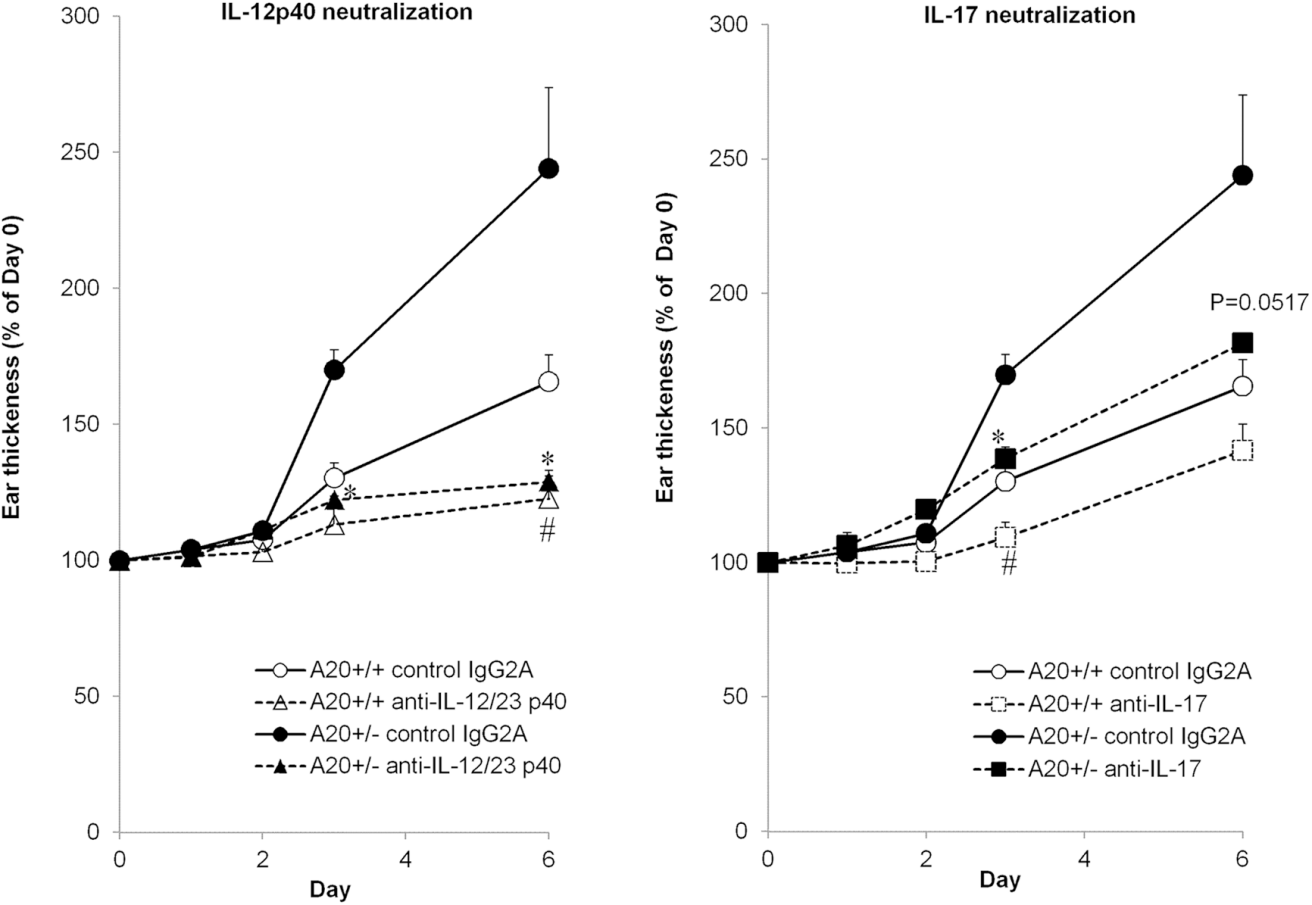
Effect of neutralization of IL-17 or IL-23 to ear inflammation in IMQ-treated A20 knockout mice. Thickness of IMQ- treated ears of male A20^+/-^ and A20^+/+^ littermate mice with 100 μg/body anti-IL-17 antibody, anti-IL-12p40 antibody, or control IgG2A antibody (R&D Systems) after 6 consecutive days. Antibodies were administrated by intravenous injection the day before the IMQ treatment. Error bars represent SEM; N = 3 or 4 for each group, #*p* < 0.05 compared to IMQ- and control IgG2A-treated A20^+/+^ mice and *p < 0.05 compared to IMQ- and anti-IL-17 antibody-treated A20^+/-^ mice by Student’s *t*-test.

IMQ-induced psoriasis involves IL-23-mediated IL-17 production [20]. As we observed increased IL-17 and IL-12p40 production in ears of IMQ-treated A20^+/-^ mice, we tested whether neutralization of IL-17 or IL-12/23p40 abrogated IMQ-induced psoriasis in haploinsufficient mice. Administration of anti-IL-12/23p40 antibody completely inhibited IMQ-induced ear swelling in A20^+/-^ mice to the same levels observed in A20^+/+^ mice. These results revealed that neutralizing the increased IL-12p40 production by A20 deficiency improved IMQ-induced skin inflammation. As IL-12p40 is a subunit of both IL-12 and IL-23, it is possible that A20 controls sensitivity to either or both these cytokines. Recently, it has been shown that A20 directly restricted IL-17RA-triggered signaling via interactions with the distal domain of IL-17RA [25]. However, administration of anti-IL-17 antibody was only partially effective in inhibiting ear swelling in both A20^+/+^ and A20^+/-^ mice (Fig 3). These data suggest that both IL-17-dependent and -independent pathways may contribute to IMQ-induced skin inflammation.

### A20 restricts IL-17 production and resultant dermatitis induced by IL-23

**Fig 4:**
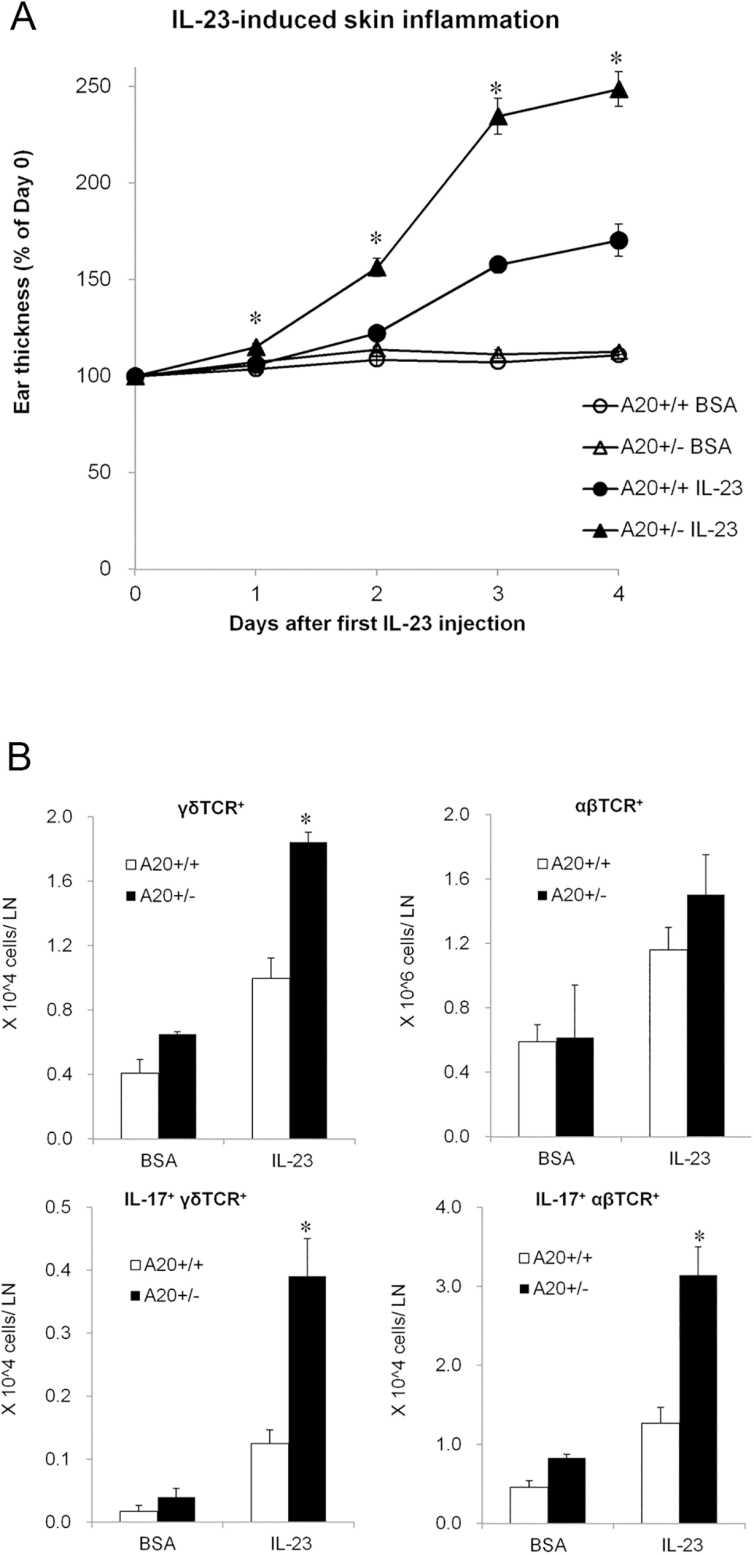
A20 restricts interleukin (IL)-23 signal to IL-17 producing cells. (A) Thickness of IL-23- or BSA-treated ears in A20^+/-^ and A20^+/+^ littermate mice. Data are representative of two experiments. Error bars represent SEM; N = 3 (male, BSA injected group) or 9 (1 male and 8 female of A20^+/-^ mice, and 2 male and 7 female of A20^+/+^ mice for IL-23-injected group) for each group,*p < 0.05 compared to IL-23-treated A20^+/+^ mice by Student’s *t*-test. (B) Numbers of TCRγδ^+^, TCRαβ^+^, IL-17^+^ TCRγδ^+^, and IL-17^+^ TCRαβ^+^ T cells from ear-draining lymph nodes from IL-23-treated or BSA-treated mice of indicated genotypes. Data are representative of two experiments. Error bars represent SEM; N = 3 (male, BSA-injected group) or 9 (1 male and 8 female of A20^+/-^ mice, and 2 male and 7 female of A20^+/+^ mice for IL-23-injected group) for each group, *p < 0.05 compared to IL-23-treated A20^+/+^ mice by Student’s *t*-test.

To determine whether A20 restricted IL-23-dependent production of IL-17, we tested whether intradermal injection of IL-23 into A20^+/-^ exacerbated skin inflammation compared to control mice. This model has previously been described to replicate phenotypes resembling human psoriasis by recruiting IL-17-producing cells to inflammatory skin lesions [26]. Intradermal injection of IL-23 for 4 consecutive days caused greater ear thickness in A20^+/-^ than in A20^+/+^ control mice (Fig 4A). FACS analyses revealed that A20^+/-^ mice showed increased numbers of IL-17-producing γδT cells and αβT cells (Fig 4B). These data suggest that A20 controls not only TLR7/8 responses but also IL-23-induced recruitment of IL-17-producing cells into inflamed tissue. Increased production of cytokines such as IL-1β, IL-6, and IL-12p40 in inflamed ears also enhances T-helper 17 (Th17) differentiation from naïve T cells [27]. IL-17-dependent skin inflammation in this mouse model partially parallels the observation that anti-IL-17 therapy is highly effective in human patients with psoriasis [28–30].

### A20 expression is low in human patients with psoriasis in both non-lesional and lesional tissues

**Fig 5:**
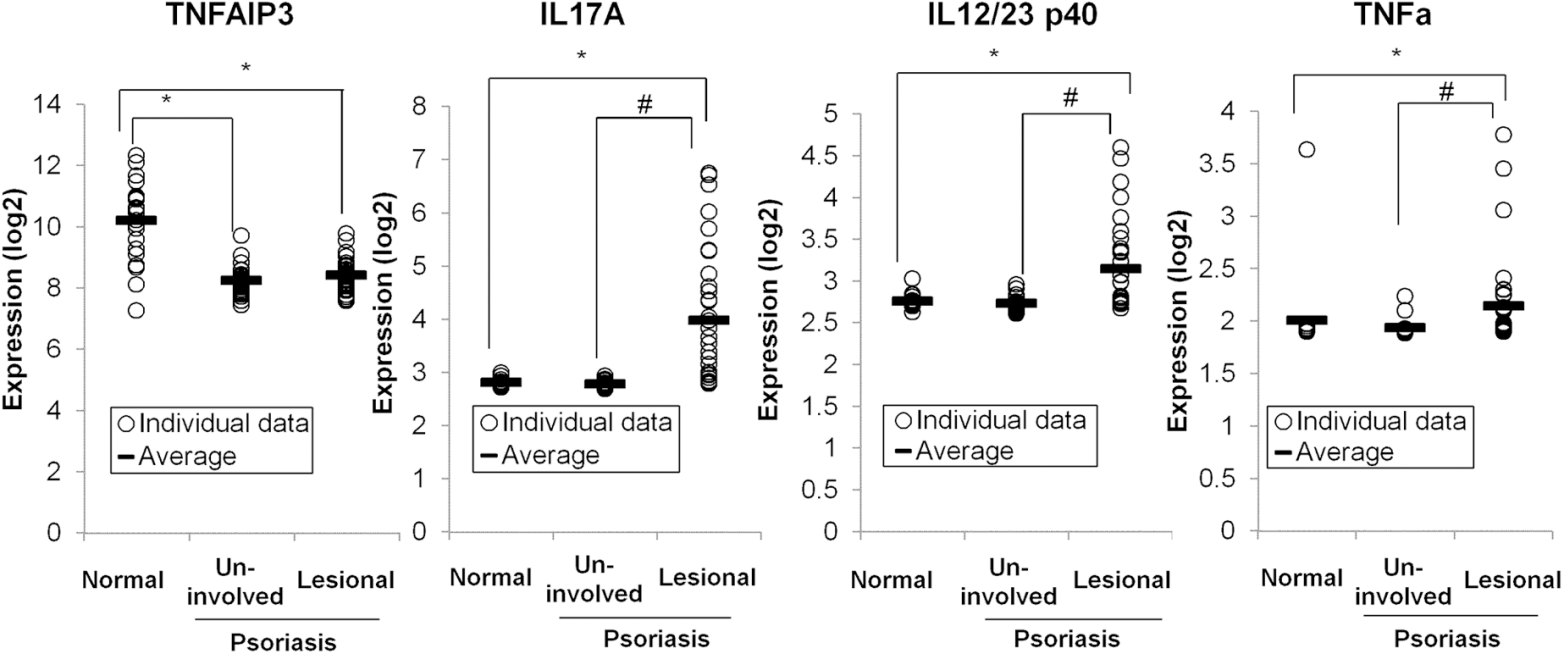
Gene expression of TNFAIP3, IL17A, IL12p40 and TNFα in psoriasis skin specimens in both non-lesional and lesional tissues. Data are obtained from GEO. DataSet type: expression profiling by array, transformed count, 82 samples, *p < 0.05 compared to normal skin of healthy controls and #*p* < 0.05 compared to uninvolved skin of patients with psoriasis by Steel’s test.

While TNFAIP3 SNPs are clearly linked to psoriasis susceptibility, the functional links between A20 expression and psoriasis susceptibility have not been elucidated. We examined gene expression data available in GEO (GEO accession no. GSE14905; [21]). This dataset includes mRNA expression data from skin biopsies from uninvolved skin and active lesions from patients with psoriasis as well as skin biopsies from healthy controls. Remarkably, TNFAIP3 expression was consistently reduced in uninvolved skin from patients with psoriasis compared to that in healthy controls (Fig 5). As reduced TNFAIP3 expression confers increased susceptibility to inflammation, this remarkable finding suggests that hypomorphic TNFAIP3 expression may be a primary driver of psoriasis susceptibility. The presumably diverse genetic background of these patients with psoriasis suggests that reduced TNFAIP3 expression may be driven by epigenetic and genetic causes. Analyses of psoriatic lesions revealed that patients with psoriasis also possessed lower TNFAIP3 expression than healthy controls (Fig 5). This result was surprising, because TNFAIP3 mRNA is an NF-κB-inducible mRNA and its level is elevated in most cell types after inflammatory stimuli (e.g., TNF and TLR). Hence, this result reinforces the notion that reduced TNFAIP3 expression may be a cause rather than a consequence of psoriatic inflammation. Psoriatic lesions contained elevated levels of IL17A and IL12/23p40 compared to uninvolved skin from patients with psoriasis, suggesting that additional epigenetic events triggering these cytokines precipitate these lesions. Zhou et al. recently described an early-onset systemic inflammation in humans caused by heterozygous germline mutations in TNFAIP3 [31]. The disease resembled Behçet's disease but not psoriasis. No apparent dermatitis occurred in A20^+/-^ mice without exogenous stimuli such as TLR7/8 ligand, suggesting the necessity of specific stimuli in addition to A20 haploinsufficiency to induce psoriasis also in humans. These human data suggest that epigenetically defined decreased TNFAIP3 expression lowers the threshold for skin inflammation in the skin of patients with psoriasis, while secondary events (e.g., microbial or trauma exposure) precipitate psoriatic lesions.

In summary, we discovered that reduced A20 expression rendered A20^+/-^ mice more susceptible to psoriasis-like skin inflammation. We showed that this susceptibility involved gene expression of IL17 and IL12/23. Moreover, our discovery of uniformly reduced A20 expression in the skin of patients with psoriasis with elevated expression of IL17A and IL12/23 p40 in lesional skin suggested that human patients with psoriasis follow a disease pathway similar to IMQ-treated A20^+/-^ mice. The role of A20 in restricting TLR responses explains why reduced A20 expression leads to increased cytokine expression [5]. In addition to prior reports indicating strong genetic associations of TNFAIP3 SNPs with psoriasis susceptibility, our current findings indicate that epigenetic events leading to uniformly reduced epidermal A20 expression are a dominant, if not universal, marker of disease. Hence, multiple pathways to psoriasis appear to proceed via reduced A20 expression. Further studies to understand the epigenetic events that affect A20 regulation are needed.

## Supporting information

S1 Fig Expression level of TNFAIP3 in bone marrow macrophages. To confirm the TNFAIP3 expression level of female A20^+/−^ and A20^+/+^ littermate control mice, expression level of TNFAIP3 in bone marrow macrophages from each genotype of mice was evaluated. To obtain bone marrow macrophages, bone marrow cells were collected from the femur of each genotype of mice and resuspended in RPMI 1640 (Thermo Fisher Scientific K.K.) containing recombinant mouse M-CSF (20 ng/mL; R&D systems) at Day 0. At Day 4 and 6, culture media were refreshed, supernatants were discarded at Day 7, and cells were stripped by TrypLE™ Express (Thermo Fisher Scientific K.K.). Cells were seeded at 5 × 10^5^ cells/mL into a 96-well plate and stimulated with lipopolysaccharide (LPS) (1 μg/mL) or IMQ (10 μg/mL) for 1, 4, and 8 h in M-CSF-containing media. After stimulation, relative mRNA expression of TNFAIP3 was quantified using 7900HT Fast Real-Time PCR System (Applied Biosystems). Circles represent the expression level of TNFAIP3 in A20^+/+^ macrophages, and triangles represent the expression level in A20^+/−^. N = 3 for each point, *p < 0.05 compared to each point of TNFAIP3 expression level in A20^+/+^ macrophages by Student’s *t*-test.
(TIF)

S2 Fig Population of splenocytes of A20^+/+^ and A20^+/−^ mice. Proportion of CD3^+^, CD19^+^, Gr-1^+^CD11b^+^, and Gr-1^-^CD11b^+^ cells in splenocytes of A20^+/+^ and A20^+/−^ mice.
(TIF)

S3 Fig Deficiency of A20 only in immune cells also aggravates ear inflammation in an IMQ-induced psoriasis model. To generate bone marrow chimeras, B6.SJL-PtprcaPepcb/BoyCrCrl (CD45.1) mice were irradiated 7 Gy twice with an X-ray irradiation system (Hitachi MBR-1520R-3, Hitachi, Ltd., Tokyo, Japan). Bone marrow cells from TNFAIP3^+/+^ or TNFAIP3^+/−^ mice (CD45.2) were injected intravenously to reconstitute the irradiated mice (2.8 × 10^6^ cells /mouse). IMQ-induced psoriasis-like dermatitis was induced at 10–14 weeks after bone marrow reconstitution. Thickness of IMQ-treated ears of male A20^+/−^ and A20^+/+^ bone marrow chimeric mice or ears of control cream (WP)-treated male B6.SJL-PtprcaPepcb/BoyCrCrl mice. Results are representative of three experiments. Error bars represent SEM; N = 2 (control group), N = 10 (IMQ-treated A20^+/−^) and N = 11 (A20^+/+^ bone marrow chimeric mice) for each group,*p < 0.05 compared to ear thickness of IMQ-treated A20^+/+^ bone marrow chimeric mice by Student’s *t*-test.
(TIF)

S1 File The ARRIVE guidelines checklist. (PDF)

S1 Table Cytokine panel of WP- or IMQ-treated ears of A20^+/+^ and A20^+/−^ mice. Cytokine concentration in ears of male A20^+/−^ and A20^+/+^ littermate mice treated with WP or IMQ for 3 or 8 consecutive days, N = 3 or 4 for each group.
(DOCX)

S2 Table Cytokine panel of IMQ-stimulated splenocytes of A20^+/+^ and A20^+/−^ mice. Cytokine concentration in IMQ-stimulated splenocytes of male A20^+/−^ and A20^+/+^ littermate mice for 1, 4, or 24 h, N = 3 for each group.
(DOCX)
