# Supplementary figures and images for: Hypomorphic A20 expression confers susceptibility to psoriasis
Source: PLoS One. 2017 Jun 28;12(6):e0180481. doi: 10.1371/journal.pone.0180481 (PMC5489224; doi:10.1371/journal.pone.0180481)

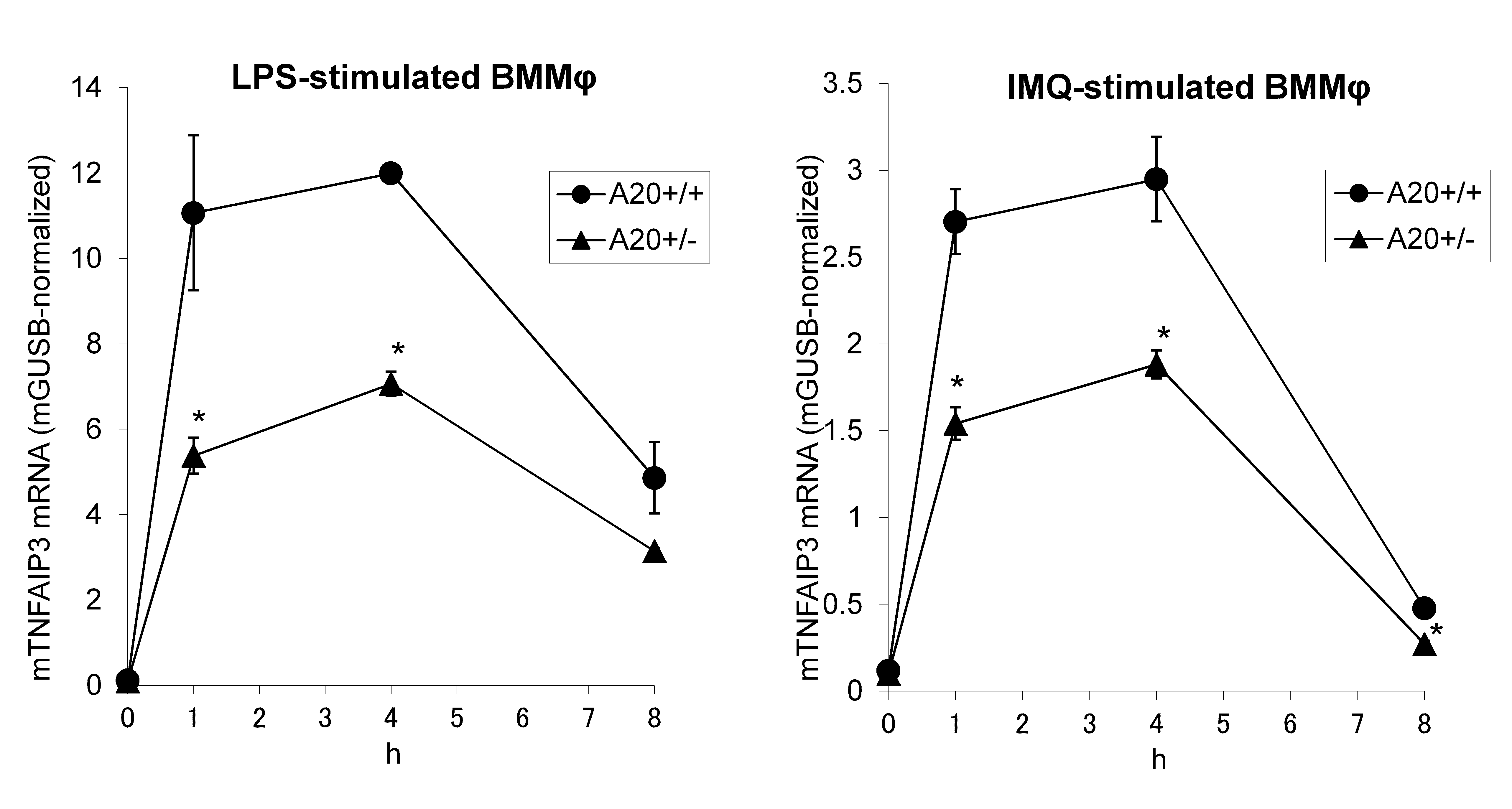

Supplement: S1 Fig — To confirm the TNFAIP3 expression level of female A20+/− and A20+/+ littermate control mice, expression level of TNFAIP3 in bone marrow macrophages from each genotype of mice was evaluated. To obtain bone marrow macrophages, bone marrow cells were collected from the femur of each genotype of mice and resuspended in RPMI 1640 (Thermo Fisher Scientific K.K.) containing recombinant mouse M-CSF (20 ng/mL; R&D systems) at Day 0. At Day 4 and 6, culture media were refreshed, supernatants were discarded at Day 7, and cells were stripped by TrypLE™ Express (Thermo Fisher Scientific K.K.). Cells were seeded at 5 × 10^5^ cells/mL into a 96-well plate and stimulated with lipopolysaccharide (LPS) (1 μg/mL) or IMQ (10 μg/mL) for 1, 4, and 8 h in M-CSF-containing media. After stimulation, relative mRNA expression of TNFAIP3 was quantified using 7900HT Fast Real-Time PCR System (Applied Biosystems). Circles represent the expression level of TNFAIP3 in A20+/+ macrophages, and triangles represent the expression level in A20+/−. N = 3 for each point, *p < 0.05 compared to each point of TNFAIP3 expression level in A20+/+ macrophages by Student’s t-test. (TIF) [file pone.0180481.s001.tif]

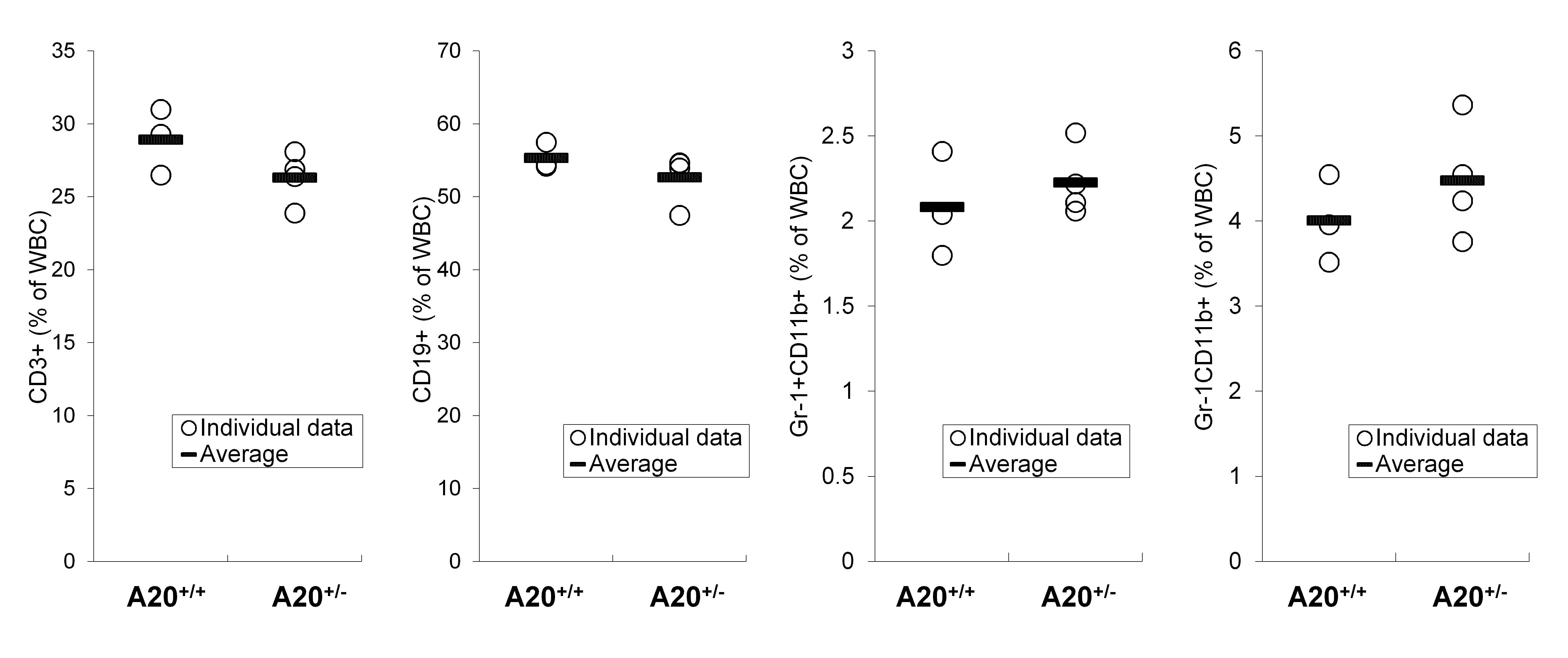

Supplement: S2 Fig — Proportion of CD3+, CD19+, Gr-1+CD11b+, and Gr-1-CD11b+ cells in splenocytes of A20+/+ and A20+/− mice. (TIF) [file pone.0180481.s002.tif]

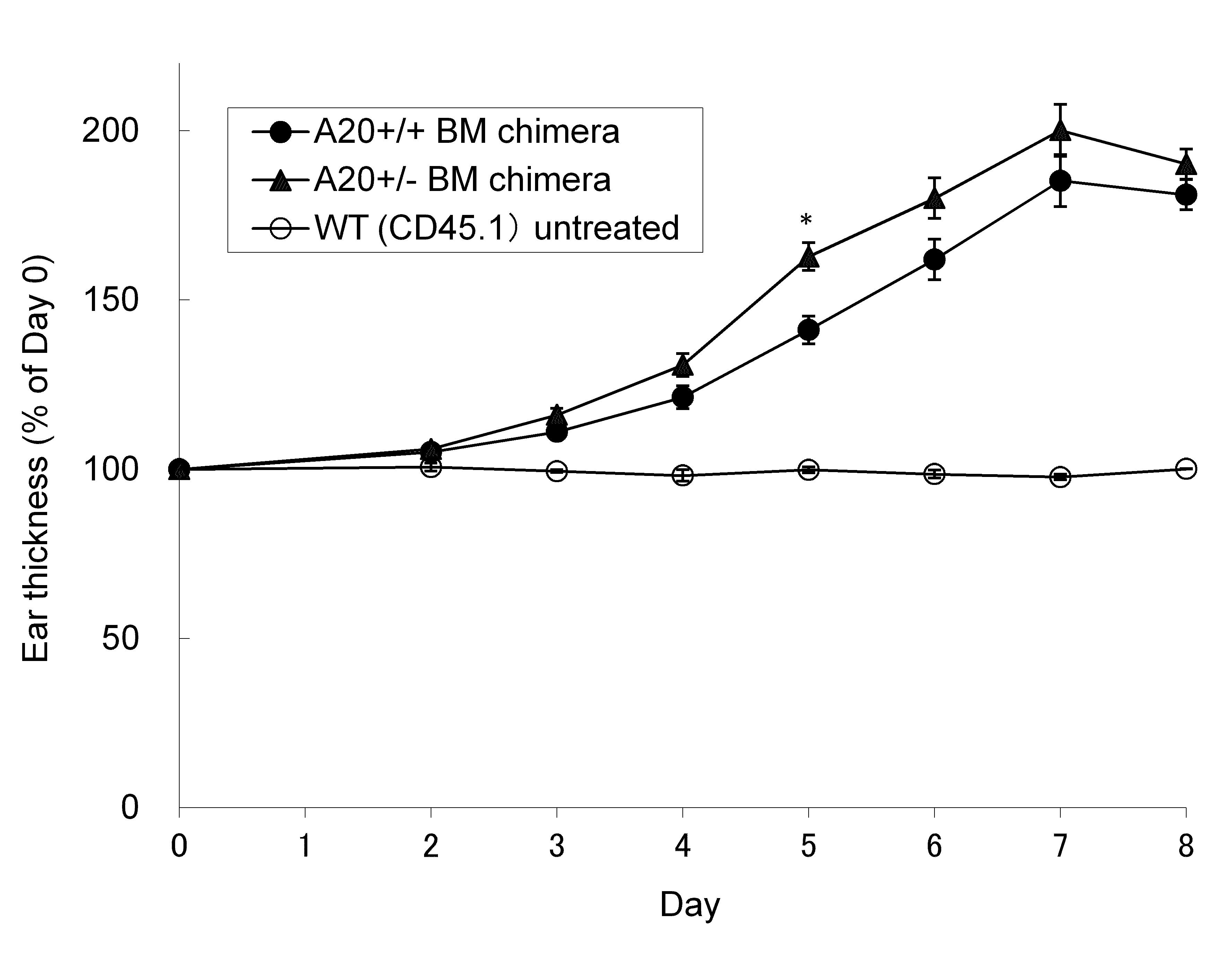

Supplement: S3 Fig — To generate bone marrow chimeras, B6.SJL-PtprcaPepcb/BoyCrCrl (CD45.1) mice were irradiated 7 Gy twice with an X-ray irradiation system (Hitachi MBR-1520R-3, Hitachi, Ltd., Tokyo, Japan). Bone marrow cells from TNFAIP3+/+ or TNFAIP3+/− mice (CD45.2) were injected intravenously to reconstitute the irradiated mice (2.8 × 10^6^ cells /mouse). IMQ-induced psoriasis-like dermatitis was induced at 10–14 weeks after bone marrow reconstitution. Thickness of IMQ-treated ears of male A20+/− and A20+/+ bone marrow chimeric mice or ears of control cream (WP)-treated male B6.SJL-PtprcaPepcb/BoyCrCrl mice. Results are representative of three experiments. Error bars represent SEM; N = 2 (control group), N = 10 (IMQ-treated A20+/−) and N = 11 (A20+/+ bone marrow chimeric mice) for each group,*p < 0.05 compared to ear thickness of IMQ-treated A20+/+ bone marrow chimeric mice by Student’s t-test. (TIF) [file pone.0180481.s003.tif]
