# Supplementary material for: Hypomorphic A20 expression confers susceptibility to psoriasis
Source: PLoS One. 2017 Jun 28;12(6):e0180481. doi: 10.1371/journal.pone.0180481 (PMC5489224; doi:10.1371/journal.pone.0180481)
Supplement: S1 Table — Cytokine concentration in ears of male A20+/− and A20+/+ littermate mice treated with WP or IMQ for 3 or 8 consecutive days, N = 3 or 4 for each group. (DOCX) [file pone.0180481.s005.docx]

**S1 Table. Cytokines panel of WP- or IMQ-treated ears of A20^+/+^ and A20^+/-^ mice .**

|  | Day3 | | | | | | | |
| --- | --- | --- | --- | --- | --- | --- | --- | --- |
|  | WP-treated | | | | IMQ-treated | | | |
|  | A20+/+ | | A20+/- | | A20+/+ | | A20+/- | |
| **cytokine  (pg/tissue)** | mean | SE | mean | SE | mean | SE | mean | SE |
| **IL-1a** | 1416.5 | 108.6 | 2100.6 | 55.4 | 2256.7 | 193.3 | 2827.2 | 53.0 |
| **IL-1b** | 252.6 | 50.8 | 305.1 | 39.6 | 3250.6 | 822.8 | 18199.4 | 1911.4 |
| **IL-2** | 39.1 | 4.2 | 43.1 | 2.0 | 83.7 | 10.6 | 138.0 | 9.7 |
| **IL-3** | 4.3 | 0.4 | 4.6 | 0.6 | 13.8 | 1.9 | 26.2 | 2.0 |
| **IL-4** | 17.1 | 3.1 | 15.8 | 0.7 | 43.9 | 2.8 | 202.2 | 81.6 |
| **IL-5** | 10.0 | 1.7 | 10.4 | 0.3 | 26.0 | 4.2 | 44.5 | 4.4 |
| **IL-6** | 19.4 | 3.2 | 19.8 | 1.2 | 74.9 | 11.3 | 456.1 | 72.0 |
| **IL-9** | Below detection limit | | | | Below detection limit | | | |
| **IL-10** | 54.8 | 5.9 | 54.8 | 2.4 | 120.8 | 15.7 | 235.9 | 20.5 |
| **IL-12p40** | 11.6 | 4.7 | 33.6 | 9.8 | 1986.4 | 373.8 | 3921.3 | 201.9 |
| **IL-12p70** | 233.6 | 8.1 | 303.9 | 13.4 | 529.5 | 55.6 | 907.0 | 70.2 |
| **IL-13** | 259.2 | 28.3 | 260.2 | 5.3 | 589.5 | 96.1 | 1457.5 | 251.7 |
| **IL-17** | 20.2 | 2.0 | 21.0 | 1.4 | 164.4 | 40.1 | 390.1 | 38.7 |
| **Eotaxin** | Below detection limit | | | | Below detection limit | | | |
| **G-CSF** | 22.2 | 8.4 | 26.6 | 9.3 | 755.3 | 210.7 | 5082.5 | 785.3 |
| **GM-CSF** | 128.0 | 12.5 | 175.6 | 24.7 | 504.7 | 51.8 | 1134.5 | 108.2 |
| **IFN-g** | Below detection limit | | | | Below detection limit | | | |
| **KC** | 14.2 | 4.2 | 27.7 | 9.0 | 1369.9 | 249.5 | 5790.8 | 625.5 |
| **MCP-1** | 121.3 | 19.3 | 123.8 | 7.8 | 850.6 | 101.3 | 2429.4 | 400.9 |
| **MIP-1a** | 32.7 | 15.8 | 44.8 | 11.2 | 394.8 | 57.3 | 2895.1 | 827.5 |
| **MIP-1b** | 131.1 | 13.0 | 164.6 | 18.3 | 301.2 | 37.4 | 632.8 | 121.6 |
| **RANTES** | 35.2 | 26.5 | 8.4 | 1.6 | 101.9 | 27.5 | 186.9 | 38.7 |
| **TNF-a** | 100.1 | 8.0 | 152.4 | 13.3 | 447.0 | 81.8 | 900.8 | 67.9 |

|  | Day8 | | | | | | | |
| --- | --- | --- | --- | --- | --- | --- | --- | --- |
|  | WP-treated | | | | IMQ-treated | | | |
|  | A20+/+ | | A20+/- | | A20+/+ | | A20+/- | |
| **cytokine  (pg/tissue)** | mean | SE | mean | SE | mean | SE | mean | SE |
| **IL-1a** | 1776.2 | 77.7 | 1546.1 | 106.2 | 4278.7 | 83.2 | 5501.8 | 227.2 |
| **IL-1b** | 408.9 | 93.1 | 649.4 | 318.9 | 1690.0 | 309.6 | 5037.9 | 887.1 |
| **IL-2** | 51.5 | 4.6 | 47.5 | 5.6 | 121.8 | 2.2 | 140.4 | 6.3 |
| **IL-3** | 6.9 | 0.4 | 4.5 | 0.6 | 17.8 | 2.1 | 22.3 | 2.1 |
| **IL-4** | 18.0 | 1.8 | 16.1 | 2.2 | 42.0 | 2.8 | 84.3 | 8.0 |
| **IL-5** | 9.1 | 1.0 | 9.3 | 0.5 | 26.7 | 1.7 | 37.7 | 4.2 |
| **IL-6** | 26.8 | 3.8 | 27.1 | 4.9 | 71.4 | 2.8 | 99.3 | 14.5 |
| **IL-9** | Below detection limit | | | | Below detection limit | | | |
| **IL-10** | 58.8 | 5.9 | 59.9 | 4.2 | 145.6 | 15.1 | 195.3 | 11.0 |
| **IL-12p40** | 43.0 | 21.5 | 26.6 | 3.9 | 1158.4 | 255.3 | 3060.9 | 361.5 |
| **IL-12p70** | 280.2 | 19.6 | 252.8 | 13.4 | 807.0 | 20.3 | 1193.9 | 84.8 |
| **IL-13** | 300.5 | 49.5 | 296.8 | 18.9 | 816.0 | 36.0 | 1152.7 | 57.7 |
| **IL-17** | 25.3 | 3.6 | 22.1 | 2.5 | 164.0 | 7.8 | 346.5 | 39.0 |
| **Eotaxin** | Below detection limit | | | | Below detection limit | | | |
| **G-CSF** | 64.1 | 31.1 | 129.4 | 102.0 | 138.8 | 30.5 | 742.0 | 106.4 |
| **GM-CSF** | 174.3 | 21.6 | 189.7 | 30.6 | 580.5 | 36.3 | 830.3 | 50.2 |
| **IFN-g** | Below detection limit | | | | Below detection limit | | | |
| **KC** | 57.3 | 25.3 | 114.9 | 70.3 | 262.8 | 45.1 | 874.2 | 270.0 |
| **MCP-1** | 128.9 | 20.2 | 181.9 | 69.0 | 398.7 | 43.6 | 677.2 | 86.3 |
| **MIP-1a** | 56.8 | 16.6 | 146.7 | 93.3 | 295.3 | 86.3 | 911.4 | 61.8 |
| **MIP-1b** | 179.4 | 23.3 | 192.2 | 18.6 | 404.2 | 60.3 | 398.2 | 29.1 |
| **RANTES** | 7.8 | 1.8 | 11.8 | 2.9 | 82.5 | 21.5 | 129.7 | 8.0 |
| **TNF-a** | 158.5 | 19.7 | 174.8 | 26.0 | 672.6 | 26.3 | 802.2 | 45.8 |
