# Supplementary material for: Hypomorphic A20 expression confers susceptibility to psoriasis
Source: PLoS One. 2017 Jun 28;12(6):e0180481. doi: 10.1371/journal.pone.0180481 (PMC5489224; doi:10.1371/journal.pone.0180481)
Supplement: S2 Table — Cytokine concentration in IMQ-stimulated splenocytes of male A20+/− and A20+/+ littermate mice for 1, 4, or 24 h, N = 3 for each group. (DOCX) [file pone.0180481.s006.docx]

**S2 Table. Cytokines panel of IMQ-stimulated splenocytes of A20^+/+^ and A20^+/-^ mice .**

|  | 0h | | | | 1h | | | |
| --- | --- | --- | --- | --- | --- | --- | --- | --- |
|  | A20+/+ | | A20+/- | | A20+/+ | | A20+/- | |
| **cytokine  (pg/tissue)** | mean | SE | mean | SE | mean | SE | mean | SE |
| **IL-1a** | Below detection limit | | | | Below detection limit | | Below detection limit | |
| **IL-1b** | 0.9 | 0.9 | Below detection limit | | Below detection limit | | 6.8 | 4.9 |
| **IL-2** | Below detection limit | | | | Below detection limit | | | |
| **IL-3** | Below detection limit | | | | Below detection limit | | | |
| **IL-4** | Below detection limit | | | | Below detection limit | | | |
| **IL-5** | Below detection limit | | | | Below detection limit | | | |
| **IL-6** | Below detection limit | | | | Below detection limit | | | |
| **IL-9** | 11.5 | 8.8 | Below detection limit | | 8.2 | 7.7 | Below detection limit | |
| **IL-10** | 0.0 | 0.0 | Below detection limit | | Below detection limit | | | |
| **IL-12p40** | 2.7 | 0.3 | 13.6 | 3.4 | 3.1 | 1.2 | 19.1 | 4.3 |
| **IL-12p70** | Below detection limit | | | | Below detection limit | | 0.6 | 0.6 |
| **IL-13** | 0.5 | 0.5 | 0.2 | 0.2 | 0.1 | 0.1 | 2.0 | 1.0 |
| **IL-17** | Below detection limit | | | | Below detection limit | | | |
| **Eotaxin** | Below detection limit | | | | 22.1 | 22.1 | Below detection limit | |
| **G-CSF** | Below detection limit | | | | Below detection limit | | | |
| **GM-CSF** | Below detection limit | | | | Below detection limit | | | |
| **IFN-g** | Below detection limit | | | | Below detection limit | | | |
| **KC** | Below detection limit | | | | Below detection limit | | 8.8 | 3.3 |
| **MCP-1** | Below detection limit | | | | Below detection limit | | | |
| **MIP-1a** | Below detection limit | | | | 1.8 | 1.8 | 13.4 | 9.8 |
| **MIP-1b** | 15.8 | 1.4 | 22.4 | 2.4 | 23.6 | 3.8 | 59.1 | 23.2 |
| **RANTES** | 9.4 | 0.4 | 11.5 | 1.4 | 15.3 | 3.9 | 33.7 | 16.9 |
| **TNF-a** | 0.3 | 0.2 | 0.0 | 0.0 | 0.7 | 0.4 | 3.0 | 0.4 |

|  | 4h | | | | 24h | | | |
| --- | --- | --- | --- | --- | --- | --- | --- | --- |
|  | A20+/+ | | A20+/- | | A20+/+ | | A20+/- | |
| **cytokine  (pg/tissue)** | mean | SE | mean | SE | mean | SE | mean | SE |
| **IL-1a** | Below detection limit | | | | Below detection limit | | | |
| **IL-1b** | 18.3 | 2.2 | 66.5 | 15.9 | 94.5 | 3.6 | 114.4 | 10.2 |
| **IL-2** | Below detection limit | | | | Below detection limit | | | |
| **IL-3** | Below detection limit | | | | Below detection limit | | | |
| **IL-4** | Below detection limit | | | | Below detection limit | | | |
| **IL-5** | Below detection limit | | | | Below detection limit | | | |
| **IL-6** | 2.5 | 0.7 | 44.6 | 24.7 | 181.8 | 5.1 | 480.7 | 123.8 |
| **IL-9** | 25.0 | 5.3 | 1.9 | 1.9 | 30.4 | 6.0 | 8.4 | 6.3 |
| **IL-10** | 0.0 | 0.0 | 1.7 | 1.6 | 11.6 | 0.8 | 26.3 | 4.4 |
| **IL-12p40** | 6.8 | 0.1 | 57.3 | 8.0 | 129.1 | 7.4 | 315.3 | 49.4 |
| **IL-12p70** | 1.8 | 0.3 | 11.4 | 5.4 | 19.5 | 1.2 | 28.4 | 5.4 |
| **IL-13** | 2.0 | 0.6 | 10.3 | 3.9 | 14.8 | 0.6 | 22.3 | 3.1 |
| **IL-17** | Below detection limit | | | | Below detection limit | | | |
| **Eotaxin** | 65.2 | 37.4 | 67.4 | 67.4 | 271.8 | 53.2 | 191.3 | 120.8 |
| **G-CSF** | Below detection limit | | | | Below detection limit | | | |
| **GM-CSF** | Below detection limit | | | | Below detection limit | | | |
| **IFN-g** | Below detection limit | | | | Below detection limit | | 25.0 | 13.9 |
| **KC** | Below detection limit | | 23.0 | 7.7 | Below detection limit | | 21.3 | 10.2 |
| **MCP-1** | Below detection limit | | 39.2 | 13.3 | 18.3 | 1.3 | 61.6 | 9.6 |
| **MIP-1a** | 39.8 | 3.0 | 325.4 | 153.9 | 658.5 | 29.1 | 1208.2 | 184.6 |
| **MIP-1b** | 93.0 | 3.5 | 559.7 | 172.1 | 958.7 | 80.6 | 1917.0 | 247.2 |
| **RANTES** | 15.2 | 0.9 | 237.4 | 167.1 | 241.6 | 14.4 | 693.9 | 251.1 |
| **TNF-a** | 6.5 | 1.3 | 66.2 | 10.5 | 7.9 | 1.4 | 21.4 | 6.8 |
